# 13-Nitro­benzo[*a*][1,4]benzo­thia­zino[3,2-*c*]phenoxazine

**DOI:** 10.1107/S2414314624002992

**Published:** 2024-04-26

**Authors:** Mamoun M. Bader, Cameron Fiester, Phuong-Truc T. Pham, Amy Bradely, Amjad Nazzal

**Affiliations:** a Department of Chemistry, College of Science, Alfasial University, Riyadh 11533, Saudi Arabia; b Department of Chemistry, Wilkes University, Wilkes Barre, PA, USA; c Department of Chemistry, Pennsylvania State University, Scranton, PA 18512, USA; d Department of Physics, Wilkes University, Wilkes Barre, PA, USA; Institute of Chemistry, Chinese Academy of Sciences

**Keywords:** crystal structure, donor/acceptor, non centrosymmetric dye, fused heterocyclic, ladder oligomer

## Abstract

In the title compound, C_22_H_11_N_3_O_3_S, dihedral angle between the phenyl rings on the periphery of the molecule is 8.05 (18)°. In the crystal, aromatic π–π stacking distance and short C—H⋯O contacts are observed. The maximum absorption occurs at 688 nm.

## Abstract 2

The title compound crystallizes in the non-centrosymmetric ortho­rhom­bic space group *Fdd*2, with 16 mol­ecules in the unit cell. In the crystal, aromatic π–π stacking distance and short C—H⋯O contacts are observed.

## Graphical abstract

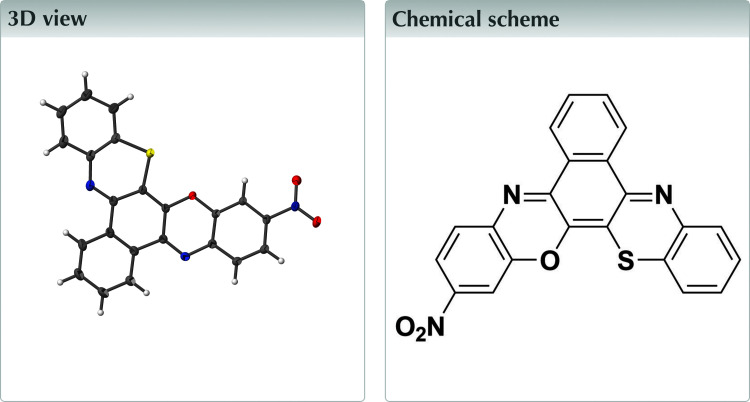

## Structure description

**Figure 1:**
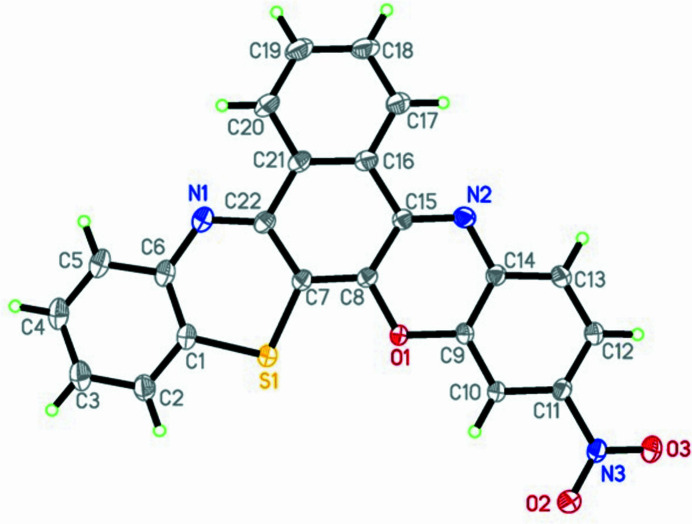
Structure of title compound.with displacement ellipsoids drawn at the 50% probability level.

One area of inter­est in fused heterocyclic aromatic compounds is their potential to act as alternatives to oligoacenes for use in organic semiconducting devices (McLean *et al.*, 1989 ▸, 1990 ▸; Pham *et al.*, 2008 ▸). Surprisingly, despite this intensely researched area, structural studies of these materials are scarce. Pheno­thia­zine systems are readily obtained by reaction of halo-*p*-benzo­quinones and amino thio­phenols (Agarwal *et al.*, 1980 ▸; Okafor *et al.*, 1988 ▸; Spangler *et al.*, 1989 ▸; Faleh *et al.*, 2008 ▸) The title compound, C_22_H_11_N_3_O_3_S, is an asymmetric mol­ecule with sulfur and oxygen bridging atoms (Fig. 1 ▸) that was prepared in two steps.

**Figure 2:**
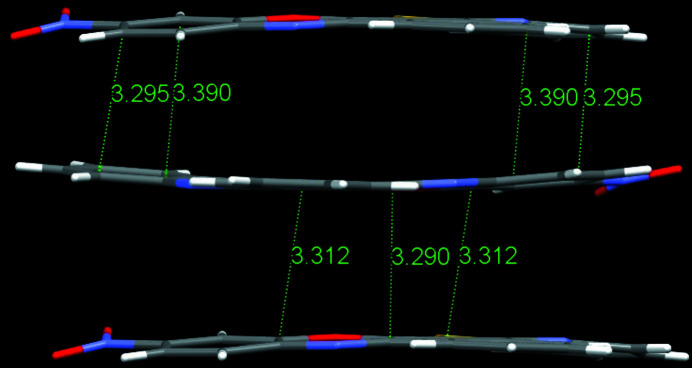
Stacking of molecules with shortest observed distances (atom to atom).

The mol­ecule is quasi-planar, as indicated by the torsion angles C8—C7—C22—N1 [−179.49 (19)°], S1—C7—C8—C15 [179.71 (16)°] and O2—N3—C11—C10 [−8.3 (3)°]. The nitro group subtends a dihedral angle of 8.0 (3)° with respect to its attached ring. A π–π-stacking distance of 3.290 (3) Å (Fig. 2 ▸) and close C—H⋯O inter­actions [H19⋯O2(1 − *x*, 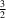 − *y*, 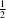 + *z*) = 2.44, H12⋯O2( 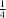 + *x*, 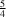 − *y*, 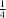 + *z*) = 2.50 and H3⋯O3( 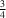 − *x*, 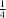 + *y*, − 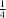 + *z*) 2.63 Å] are observed.

A survey of the Cambridge Structural Database (Groom *et al.*, 2016 ▸) on March 28, 2024 revealed no hits for this compound or any closely related structure. The closest is a structure from our group of the symmetrical mol­ecule 15,16-di­thia-5,10-di­aza­naphtho­[2,3-*a*]benzo[*c*]anthracene, (Pham *et al.*, 2008 ▸), which crystallizes in the monoclinic space group *P*2_1_/*c* with four mol­ecules in the unit cell. This mol­ecule is also quasi-planar with dihedral angles between the three phenyl rings on the periphery of the mol­ecule ranging from 1.89 to 6.65° and close C—H⋯S and C—H⋯N contacts. In comparison, no C—H⋯S or C—H⋯N close contacts are observed in the title compound.

## Synthesis and crystallization

**Figure 3:**
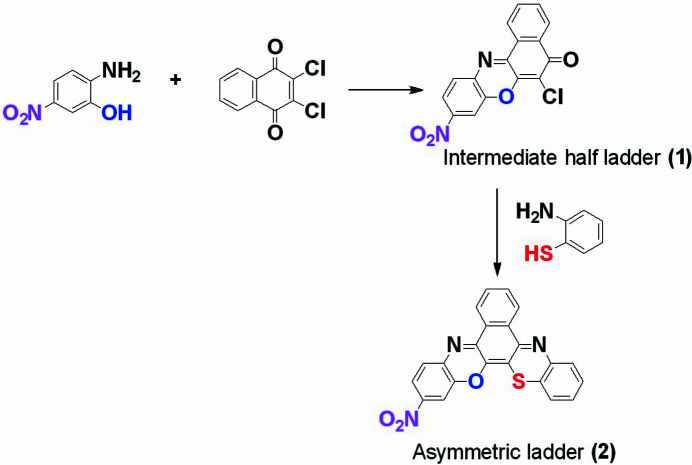
Reaction scheme for the preparation of the title compound.

The target mol­ecule was synthesized in a two-step process following published procedures (Agarwal *et al.*, 1980 ▸; Okafor *et al.*, 1988 ▸; Fiester *et al.*, 2023 ▸; Spangler *et al.*, 1989 ▸) as shown in Fig. 3 ▸.

*Synthesis of the precursor 6-chloro-9-nitro-5-oxo-5H-benzo[a]phenoxazine (**1**)*

2-Amino-5-nitro­phenol (5 mmol, 0.7612 g) and potassium acetate (5 mmol, 0.7740 g) were combined in 25 mL of ethanol. 2,3-Di­chloro-1,4-napthophenol (5 mmol, 1.120 g) was added to the solution, which was then heated gently for 2 h. The solution was cooled and filtered, resulting in an orange–red solid (1.0028 g, 77%), m.p. 255°C.

*Synthesis of the title compound 13-nitro­benzo[a][1,4]benzo­thia­zino[3,2-c]phenoxazine*

2-Amino­thio­phenol (excess) and potassium acetate were combined in 10 mL of ethanol. Precursor **1** (0.9781 g, 3.7 mmol) was added to the solution, which was then heated at 60°C for approximately 5 h. The solution was cooled and filtered, resulting in a dark-blue solid (0.4136 g, 33% yield), m.p. 340°C. The product was crystallized by slow evaporation from di­chloro­methane solution.

## Refinement

Crystal data, data collection and structure refinement details are summarized in Table 1 ▸.

## Supplementary Material

Crystal structure: contains datablock(s) I. DOI: 10.1107/S2414314624002992/nx4001sup1.cif

Structure factors: contains datablock(s) I. DOI: 10.1107/S2414314624002992/nx4001Isup2.hkl

Supporting information file. DOI: 10.1107/S2414314624002992/nx4001Isup3.cml

CCDC reference: 2347503

Additional supporting information:  crystallographic information; 3D view; checkCIF report

## Figures and Tables

**Table 1:** Experimental details

Crystal data	
Chemical formula	C_22_H_11_N_3_O_3_S
*M*_r_	397.40
Crystal system, space group	Orthorhombic, *F* *d* *d*2
Temperature (K)	130
*a*, *b*, *c* (Å)	6.7497 (9), 52.478 (7), 18.832 (3)
*V* (Å^3^)	6670.5 (16)
*Z*	16
Radiation type	Mo *K*α
μ (mm^−1^)	0.23
Crystal size (mm)	0.20 × 0.13 × 0.05

Data collection	
Diffractometer	Bruker PHOTON-III CPAD
Absorption correction	Multi-scan (*SADABS*; Krause *et al.*, 2015 ▸)
*T*_min_, *T*_max_	0.681, 0.746
No. of measured, independent and observed [*I* > 2σ(*I*)] reflections	22321, 5093, 4718
*R*_int_	0.034
(sin θ/λ)_max_ (Å^−1^)	0.714

Refinement	
*R*[*F* ^2^ > 2σ(*F* ^2^)], *wR*(*F* ^2^), *S*	0.033, 0.087, 1.03
No. of reflections	5093
No. of parameters	262
No. of restraints	1
H-atom treatment	H-atom parameters constrained
Δρ_max_, Δρ_min_ (e Å^−3^)	0.36, −0.23
Absolute structure	Flack *x* determined using 2109 quotients [(*I* ^+^)−(*I* ^−^)]/[(*I* ^+^)+(*I* ^−^)] (Parsons *et al.*, 2013 ▸)
Absolute structure parameter	0.01 (3)

Computer programs: *APEX4* and *SAINT* (Bruker, 2014 ▸), *SHELXT2018/2* (Sheldrick, 2015*a* ▸), *SHELXL2018/3* (Sheldrick, 2015*b* ▸) and *SHELXTL* (Sheldrick, 2008 ▸).

## full crystallographic data

### Crystal data

**Table:**

C_22_H_11_N_3_O_3_S	*D*_x_ = 1.583 Mg m^−^^3^
*M**_r_* = 397.40	Mo *K*α radiation, λ = 0.71073 Å
Orthorhombic, *F**d**d*2	Cell parameters from 2857 reflections
*a* = 6.7497 (9) Å	θ = 2.3–30.0°
*b* = 52.478 (7) Å	µ = 0.23 mm^−^^1^
*c* = 18.832 (3) Å	*T* = 130 K
*V* = 6670.5 (16) Å^3^	Needle, brown
*Z* = 16	0.20 × 0.13 × 0.05 mm
*F*(000) = 3264	

### Data collection

**Table:**

Bruker PHOTON-III CPAD diffractometer	4718 reflections with *I* > 2σ(*I*)
Radiation source: micro-focus	*R*_int_ = 0.034
φ and ω scans	θ_max_ = 30.5°, θ_min_ = 2.3°
Absorption correction: multi-scan (SADABS; Krause *et al.*, 2015)	*h* = −6→9
*T*_min_ = 0.681, *T*_max_ = 0.746	*k* = −74→73
22321 measured reflections	*l* = −26→26
5093 independent reflections	

### Refinement

**Table:**

Refinement on *F*^2^	Hydrogen site location: inferred from neighbouring sites
Least-squares matrix: full	H-atom parameters constrained
*R*[*F*^2^ > 2σ(*F*^2^)] = 0.033	*w* = 1/[σ^2^(*F*_o_^2^) + (0.0596*P*)^2^] where *P* = (*F*_o_^2^ + 2*F*_c_^2^)/3
*wR*(*F*^2^) = 0.087	(Δ/σ)_max_ = 0.003
*S* = 1.03	Δρ_max_ = 0.36 e Å^−^^3^
5093 reflections	Δρ_min_ = −0.23 e Å^−^^3^
262 parameters	Absolute structure: Flack *x* determined using 2109 quotients [(*I*^+^)-(*I*^-^)]/[(*I*^+^)+(*I*^-^)] (Parsons *et al.*, 2013)
1 restraint	Absolute structure parameter: 0.01 (3)

### Special details

**Table:**

Experimental. Prof. M. Bader/C. Fiester
Geometry. All esds (except the esd in the dihedral angle between two l.s. planes) are estimated using the full covariance matrix. The cell esds are taken into account individually in the estimation of esds in distances, angles and torsion angles; correlations between esds in cell parameters are only used when they are defined by crystal symmetry. An approximate (isotropic) treatment of cell esds is used for estimating esds involving l.s. planes.

### Fractional atomic coordinates and isotropic or equivalent isotropic displacement parameters (Å^2^)

**Table:**

	*x*	*y*	*z*	*U*_iso_*/*U*_eq_	
S1	0.51936 (8)	0.77762 (2)	0.36455 (3)	0.02344 (12)	
O1	0.4662 (2)	0.73153 (2)	0.43558 (7)	0.0198 (3)	
O2	0.3593 (2)	0.64760 (3)	0.32680 (8)	0.0293 (3)	
O3	0.4057 (3)	0.62015 (3)	0.41134 (8)	0.0338 (4)	
N1	0.5694 (3)	0.82016 (3)	0.48286 (9)	0.0227 (3)	
N2	0.4745 (3)	0.72228 (3)	0.58474 (9)	0.0209 (3)	
N3	0.3919 (3)	0.64213 (3)	0.38935 (9)	0.0225 (3)	
C1	0.5695 (3)	0.81010 (4)	0.35380 (11)	0.0223 (4)	
C2	0.5942 (3)	0.81878 (4)	0.28432 (12)	0.0273 (4)	
H2	0.581985	0.807310	0.245538	0.033*	
C3	0.6371 (3)	0.84436 (4)	0.27226 (13)	0.0312 (4)	
H3	0.655028	0.850328	0.225073	0.037*	
C4	0.6537 (3)	0.86123 (4)	0.32886 (13)	0.0306 (5)	
H4	0.683467	0.878653	0.320257	0.037*	
C5	0.6269 (3)	0.85268 (4)	0.39781 (13)	0.0271 (4)	
H5	0.637067	0.864410	0.436066	0.033*	
C6	0.5849 (3)	0.82691 (4)	0.41212 (11)	0.0227 (4)	
C7	0.5201 (3)	0.77476 (3)	0.45674 (10)	0.0189 (3)	
C8	0.4934 (3)	0.75094 (3)	0.48421 (10)	0.0180 (3)	
C9	0.4496 (3)	0.70709 (3)	0.46210 (9)	0.0177 (3)	
C10	0.4258 (3)	0.68769 (3)	0.41332 (9)	0.0181 (3)	
H10	0.416843	0.691018	0.363837	0.022*	
C11	0.4155 (3)	0.66298 (3)	0.44028 (10)	0.0198 (3)	
C12	0.4311 (3)	0.65728 (4)	0.51177 (10)	0.0225 (4)	
H12	0.429063	0.640118	0.527818	0.027*	
C13	0.4499 (3)	0.67737 (4)	0.55959 (10)	0.0223 (4)	
H13	0.457868	0.673908	0.609021	0.027*	
C14	0.4573 (3)	0.70262 (4)	0.53568 (10)	0.0192 (4)	
C15	0.4910 (3)	0.74534 (4)	0.55893 (10)	0.0184 (3)	
C16	0.5132 (3)	0.76694 (4)	0.60727 (11)	0.0212 (4)	
C17	0.5121 (3)	0.76263 (4)	0.68082 (11)	0.0283 (4)	
H17	0.492764	0.745872	0.698569	0.034*	
C18	0.5391 (4)	0.78256 (5)	0.72745 (12)	0.0370 (5)	
H18	0.536487	0.779604	0.777212	0.044*	
C19	0.5703 (4)	0.80725 (5)	0.70113 (12)	0.0355 (5)	
H19	0.591278	0.820946	0.733259	0.043*	
C20	0.5707 (4)	0.81181 (4)	0.62893 (11)	0.0280 (4)	
H20	0.591466	0.828616	0.611690	0.034*	
C21	0.5408 (3)	0.79174 (4)	0.58094 (10)	0.0214 (4)	
C22	0.5444 (3)	0.79669 (4)	0.50355 (10)	0.0202 (3)	

### Atomic displacement parameters (Å^2^)

**Table:**

	*U*^11^	*U*^22^	*U*^33^	*U*^12^	*U*^13^	*U*^23^
S1	0.0338 (3)	0.01553 (18)	0.02103 (19)	−0.00169 (18)	0.00096 (19)	0.00055 (17)
O1	0.0289 (7)	0.0131 (5)	0.0174 (6)	−0.0013 (5)	−0.0005 (5)	0.0009 (5)
O2	0.0431 (9)	0.0224 (7)	0.0225 (7)	−0.0040 (7)	−0.0027 (6)	−0.0007 (5)
O3	0.0555 (10)	0.0147 (6)	0.0313 (8)	−0.0026 (7)	0.0011 (7)	0.0011 (6)
N1	0.0214 (8)	0.0178 (7)	0.0289 (8)	0.0004 (6)	−0.0017 (7)	−0.0013 (6)
N2	0.0239 (8)	0.0210 (8)	0.0178 (7)	0.0016 (6)	−0.0014 (6)	0.0003 (6)
N3	0.0279 (9)	0.0159 (7)	0.0236 (7)	−0.0029 (6)	0.0024 (7)	−0.0003 (6)
C1	0.0208 (9)	0.0172 (8)	0.0289 (10)	−0.0003 (7)	−0.0003 (7)	0.0031 (7)
C2	0.0299 (10)	0.0229 (9)	0.0291 (10)	−0.0020 (8)	−0.0007 (8)	0.0042 (7)
C3	0.0312 (11)	0.0244 (9)	0.0380 (11)	−0.0032 (8)	−0.0025 (9)	0.0089 (8)
C4	0.0281 (11)	0.0201 (9)	0.0437 (12)	−0.0024 (8)	−0.0029 (9)	0.0073 (8)
C5	0.0230 (10)	0.0175 (8)	0.0409 (11)	0.0015 (7)	−0.0027 (8)	−0.0002 (8)
C6	0.0182 (8)	0.0175 (8)	0.0323 (10)	0.0002 (7)	−0.0011 (8)	0.0011 (7)
C7	0.0193 (9)	0.0164 (8)	0.0209 (8)	0.0007 (6)	−0.0008 (6)	−0.0012 (6)
C8	0.0185 (8)	0.0166 (8)	0.0190 (8)	0.0012 (6)	−0.0009 (6)	−0.0022 (6)
C9	0.0192 (8)	0.0147 (7)	0.0191 (8)	0.0010 (6)	−0.0004 (6)	0.0022 (6)
C10	0.0215 (8)	0.0145 (7)	0.0185 (7)	−0.0005 (6)	0.0001 (7)	0.0009 (6)
C11	0.0214 (9)	0.0154 (7)	0.0226 (8)	−0.0013 (7)	0.0007 (7)	−0.0011 (6)
C12	0.0276 (9)	0.0174 (8)	0.0223 (9)	0.0000 (7)	−0.0002 (7)	0.0030 (6)
C13	0.0277 (10)	0.0198 (9)	0.0193 (8)	0.0007 (7)	−0.0003 (7)	0.0030 (6)
C14	0.0202 (8)	0.0176 (8)	0.0198 (8)	0.0008 (6)	−0.0003 (6)	−0.0002 (6)
C15	0.0183 (8)	0.0177 (8)	0.0193 (8)	0.0017 (6)	−0.0015 (6)	−0.0005 (6)
C16	0.0218 (9)	0.0215 (8)	0.0202 (8)	0.0026 (7)	−0.0026 (7)	−0.0031 (7)
C17	0.0381 (11)	0.0242 (10)	0.0227 (9)	0.0014 (8)	−0.0020 (8)	−0.0032 (8)
C18	0.0572 (15)	0.0331 (11)	0.0208 (9)	0.0032 (11)	−0.0047 (10)	−0.0081 (9)
C19	0.0500 (14)	0.0286 (11)	0.0280 (10)	0.0029 (10)	−0.0051 (10)	−0.0108 (8)
C20	0.0322 (11)	0.0218 (9)	0.0302 (10)	0.0005 (8)	−0.0026 (8)	−0.0082 (8)
C21	0.0202 (9)	0.0198 (8)	0.0244 (9)	0.0032 (7)	−0.0022 (7)	−0.0049 (7)
C22	0.0178 (8)	0.0184 (8)	0.0242 (8)	0.0011 (6)	−0.0006 (6)	−0.0028 (6)

### Geometric parameters (Å, º)

**Table:**

S1—C7	1.743 (2)	C8—C15	1.437 (2)
S1—C1	1.750 (2)	C9—C10	1.381 (2)
O1—C9	1.381 (2)	C9—C14	1.407 (2)
O1—C8	1.382 (2)	C10—C11	1.395 (2)
O2—N3	1.232 (2)	C10—H10	0.9500
O3—N3	1.229 (2)	C11—C12	1.383 (3)
N1—C22	1.303 (3)	C12—C13	1.392 (3)
N1—C6	1.382 (3)	C12—H12	0.9500
N2—C15	1.309 (2)	C13—C14	1.400 (3)
N2—C14	1.390 (2)	C13—H13	0.9500
N3—C11	1.464 (2)	C15—C16	1.461 (3)
C1—C2	1.396 (3)	C16—C17	1.403 (3)
C1—C6	1.412 (3)	C16—C21	1.405 (3)
C2—C3	1.392 (3)	C17—C18	1.378 (3)
C2—H2	0.9500	C17—H17	0.9500
C3—C4	1.390 (3)	C18—C19	1.403 (4)
C3—H3	0.9500	C18—H18	0.9500
C4—C5	1.385 (3)	C19—C20	1.381 (3)
C4—H4	0.9500	C19—H19	0.9500
C5—C6	1.408 (3)	C20—C21	1.403 (3)
C5—H5	0.9500	C20—H20	0.9500
C7—C8	1.365 (2)	C21—C22	1.480 (3)
C7—C22	1.459 (3)		
			
C7—S1—C1	101.44 (9)	C12—C11—C10	123.48 (17)
C9—O1—C8	117.11 (14)	C12—C11—N3	118.98 (16)
C22—N1—C6	122.71 (17)	C10—C11—N3	117.52 (16)
C15—N2—C14	116.53 (17)	C11—C12—C13	118.21 (18)
O3—N3—O2	123.65 (18)	C11—C12—H12	120.9
O3—N3—C11	118.20 (17)	C13—C12—H12	120.9
O2—N3—C11	118.16 (17)	C12—C13—C14	120.79 (18)
C2—C1—C6	121.09 (18)	C12—C13—H13	119.6
C2—C1—S1	116.71 (16)	C14—C13—H13	119.6
C6—C1—S1	122.20 (15)	N2—C14—C13	119.46 (17)
C3—C2—C1	119.5 (2)	N2—C14—C9	122.28 (17)
C3—C2—H2	120.2	C13—C14—C9	118.26 (17)
C1—C2—H2	120.2	N2—C15—C8	123.61 (17)
C4—C3—C2	120.4 (2)	N2—C15—C16	119.62 (18)
C4—C3—H3	119.8	C8—C15—C16	116.75 (17)
C2—C3—H3	119.8	C17—C16—C21	119.87 (19)
C5—C4—C3	120.12 (19)	C17—C16—C15	119.31 (19)
C5—C4—H4	119.9	C21—C16—C15	120.80 (18)
C3—C4—H4	119.9	C18—C17—C16	120.4 (2)
C4—C5—C6	121.1 (2)	C18—C17—H17	119.8
C4—C5—H5	119.4	C16—C17—H17	119.8
C6—C5—H5	119.4	C17—C18—C19	119.7 (2)
N1—C6—C5	116.49 (18)	C17—C18—H18	120.1
N1—C6—C1	125.70 (17)	C19—C18—H18	120.1
C5—C6—C1	117.77 (19)	C20—C19—C18	120.6 (2)
C8—C7—C22	120.54 (18)	C20—C19—H19	119.7
C8—C7—S1	117.13 (14)	C18—C19—H19	119.7
C22—C7—S1	122.32 (14)	C19—C20—C21	120.3 (2)
C7—C8—O1	116.19 (16)	C19—C20—H20	119.9
C7—C8—C15	124.04 (17)	C21—C20—H20	119.9
O1—C8—C15	119.76 (16)	C20—C21—C16	119.16 (19)
C10—C9—O1	116.98 (16)	C20—C21—C22	120.02 (19)
C10—C9—C14	122.47 (16)	C16—C21—C22	120.79 (17)
O1—C9—C14	120.55 (16)	N1—C22—C7	125.41 (18)
C9—C10—C11	116.70 (16)	N1—C22—C21	117.54 (18)
C9—C10—H10	121.6	C7—C22—C21	117.06 (17)
C11—C10—H10	121.6		
			
C7—S1—C1—C2	175.38 (16)	C12—C13—C14—N2	179.45 (19)
C7—S1—C1—C6	−4.46 (19)	C12—C13—C14—C9	−1.4 (3)
C6—C1—C2—C3	0.7 (3)	C10—C9—C14—N2	−177.73 (18)
S1—C1—C2—C3	−179.14 (16)	O1—C9—C14—N2	2.4 (3)
C1—C2—C3—C4	−0.5 (3)	C10—C9—C14—C13	3.1 (3)
C2—C3—C4—C5	−0.2 (3)	O1—C9—C14—C13	−176.79 (17)
C3—C4—C5—C6	0.7 (3)	C14—N2—C15—C8	−0.4 (3)
C22—N1—C6—C5	−175.21 (19)	C14—N2—C15—C16	−178.93 (16)
C22—N1—C6—C1	2.4 (3)	C7—C8—C15—N2	−176.72 (19)
C4—C5—C6—N1	177.30 (19)	O1—C8—C15—N2	3.7 (3)
C4—C5—C6—C1	−0.5 (3)	C7—C8—C15—C16	1.9 (3)
C2—C1—C6—N1	−177.8 (2)	O1—C8—C15—C16	−177.73 (16)
S1—C1—C6—N1	2.0 (3)	N2—C15—C16—C17	−1.3 (3)
C2—C1—C6—C5	−0.2 (3)	C8—C15—C16—C17	−179.96 (19)
S1—C1—C6—C5	179.61 (15)	N2—C15—C16—C21	176.93 (18)
C1—S1—C7—C8	−177.22 (15)	C8—C15—C16—C21	−1.7 (3)
C1—S1—C7—C22	3.86 (19)	C21—C16—C17—C18	−0.3 (3)
C22—C7—C8—O1	178.25 (16)	C15—C16—C17—C18	177.9 (2)
S1—C7—C8—O1	−0.7 (2)	C16—C17—C18—C19	−0.8 (4)
C22—C7—C8—C15	−1.4 (3)	C17—C18—C19—C20	1.2 (4)
S1—C7—C8—C15	179.71 (16)	C18—C19—C20—C21	−0.3 (4)
C9—O1—C8—C7	176.59 (17)	C19—C20—C21—C16	−0.9 (3)
C9—O1—C8—C15	−3.8 (2)	C19—C20—C21—C22	−179.1 (2)
C8—O1—C9—C10	−178.95 (17)	C17—C16—C21—C20	1.2 (3)
C8—O1—C9—C14	1.0 (2)	C15—C16—C21—C20	−177.03 (19)
O1—C9—C10—C11	178.00 (16)	C17—C16—C21—C22	179.4 (2)
C14—C9—C10—C11	−1.9 (3)	C15—C16—C21—C22	1.1 (3)
C9—C10—C11—C12	−1.1 (3)	C6—N1—C22—C7	−3.1 (3)
C9—C10—C11—N3	−179.50 (16)	C6—N1—C22—C21	176.77 (18)
O3—N3—C11—C12	−6.8 (3)	C8—C7—C22—N1	−179.49 (19)
O2—N3—C11—C12	173.20 (19)	S1—C7—C22—N1	−0.6 (3)
O3—N3—C11—C10	171.68 (18)	C8—C7—C22—C21	0.6 (3)
O2—N3—C11—C10	−8.3 (3)	S1—C7—C22—C21	179.50 (15)
C10—C11—C12—C13	2.7 (3)	C20—C21—C22—N1	−2.3 (3)
N3—C11—C12—C13	−178.88 (18)	C16—C21—C22—N1	179.57 (18)
C11—C12—C13—C14	−1.4 (3)	C20—C21—C22—C7	177.60 (19)
C15—N2—C14—C13	176.55 (18)	C16—C21—C22—C7	−0.5 (3)
C15—N2—C14—C9	−2.6 (3)		
